# The Treatment of Sprains and Fractures in the Neighbourhood of Joints by Massage and Early Passive Movements

**Published:** 1895-06-22

**Authors:** A. G. Miller

**Affiliations:** Surgeon to the Edinburgh Royal Infirmary, Lecturer on Clinical Surgery


### MEDICAL PROGRESS AND HOSPITAL CLINICS.

[*The Editor will be glad to receive offers of co-operation and contributions from members of the profession. All letters should be addressed to* THE EDITOR, THE LODGE, PORCHESTER SQUARE, LONDON, W.]

### THE TREATMENT OF SPRAINS AND FRACTURES IN THE NEIGHBOURHOOD OF JOINTS BY MASSAGE AND EARLY PASSIVE MOVEMENT.

By A. G. MILLER, M.D., F.R.C.S.E., Surgeon to the Edinburgh Royal Infirmary, Lecturer on Clinical Surgery.

Sprains have been treated by massage for many years, ever since the days of "rubbing," but only in the later stages of the treatment to remove the stiffness and swelling that persisted after the injury. In writing the article on "Sprain" for Heath's Surgical Dictionary, about ten years ago, I advocated the early employment of massage for slight sprains, and later for severe ones, because I did not then know from experience that massage may be useful from the beginning in severe as well as slight sprains. I have now no hesitation in saying that massage, properly, *i.e.*, carefully, and scientifically applied, is the best method of treatment in all sprains; and I am largely indebted to my friend, Dr. J. H. A. Laing, for this knowledge and experience.

If a sprain be seen at once, or very soon after the receipt of the injury, I would apply cold—continuous cold—to the part for twelve or twenty-four hours. Pounded ice in a waterproof bag is best, failing that, cold water; failing the cold a firm, even bandage may be applied for the purpose of preventing swelling and extravasation. After twelve or, at most, twenty-four hours have elapsed, massage should be commenced. The limb above the seat of the injury should first be stroked upwards (not rubbed) gently, the operator beginning well above the injured part, and gradually getting nearer to it. Then the swollen and discoloured part should be gently kneaded and stroked upwards, pressure being always lighter on tender parts, than on those less sensitive. After this, gentle, passive movements should be made of all joints in the neighbourhood of the injured part, including the one most damaged. This operation may take ten or fifteen minutes at first, and may be performed once a day to begin with, and after it is over a bandage or splint may be applied. After a day or two the massage may be more frequent or more prolonged, and the patient should be made to move the joint, though not necessarily to use it, the surgeon being always guided by the condition of the patient and the injured parts, everything being done with the utmost gentleness, and never in such a way as to cause harm.

The exact treatment of each case will, of course, depend on many things—the part injured, the extent of the injury, the condition of the patient, the nature of the patient's surroundings, and so on. The surgeon must, of course, in each case judge what is the most necessary and appropriate method of treatment, due prominence being always given to the main object of the treatment, viz., as Dr. Laing puts it, to make the limb useful again as soon as possible. Time has always been admitted as equal to money in the case of doctors; it is generally the same in the case of our patients' and, perhaps, even more so.

Let me give some examples in a general way. The commonest form of sprain that we have to treat is so called sprain of the ankle. It is evident that rest from use of the joint is an essential part of the treatment, at least in severe cases. One would, therefore, place the patient in bed, and put on some form of support, which latter should be capable of being taken off and reapplied easily to let the massage be performed. In the case of the wrist, on the other hand, rest in bed would not be necessary, and a rigid support would not be advisable, for stiffness is more liable to occur and to persist. Strain of the knee again, with synovitis, must be treated as a synovitis mainly, *i.e.*, by perfect rest for about a week till all fluid has disappeared. Gentle massage may be employed during that period, but rest is the main indication. Movement then is the main part of the treatment in a wrist sprain, while in the knee it is not so in my opinion.

That massage and passive movement are very useful also in the treatment of fractures in which stiffness remains after they have united, is admitted by all. But would it not be better to prevent stiffness than to have to treat it, however skilfully or successfully? Is it possible to prevent stiffness after fractures? Perhaps not, but I think that hitherto we have been too much afraid of movement, and have had too much faith in rigid apparatus.

The fractures that most frequently cause anchylosis of the neighbouring joint are those at the elbow. In regard to the treatment of these fractures there is a great difference of opinion at present. Some have perfect faith in rigid splints kept on for weeks, while others believe in early passive movement, while both sets of surgeons claim to have the most satisfactory results. For myself, I confess that I have had good and bad results with both methods.

Fractures about the ankle, again, Pott's fracture &c., are often followed by great and persistent stiffness, due largely, I believe, to long maintenance of the injured parts in one position. It is said that an uninjured joint, if kept fixed in one position for a week or two, will not become anchylosed; any stiffness will quickly disappear with gentle use. This is quite true; but it does not apply to most cases of fracture near a joint, because generally the joint is more or less injured by the same force which fractures the bone; witness the effusion into the joint, and discoloration around it, that usually occur even when the fracture does not actually extend into the joint. The stiffness, then, that results in the cases which we are considering arises from injury to the joint (followed by more or less synovitis) or the ligaments and parts around the joint (sprain). We have, then, in a case of injury near a joint, usually a double injury to deal with, viz., a fractured bone and a damaged joint. This certainly occurs in fractures near the elbow, ankle, and wrist, the most common injuries, that are apt to be followed by stiffness.

Now let me take an example from any joint. On examining the part, we find swelling, perhaps effusion into the joint, displacement, and, of course, inability to use the limb. There is also pain. Experience has taught us (the experience of our forefathers and teachers) that the first and main thing is to place the fractured surfaces of bone in position, and to maintain them so comfortably. When once we have got the displaced fragments into position we are generally quite contented that we have done a good job, and we are very much afraid of touching it again lest displacement should recur. We cover up the fracture in splints and bandages, and are afraid to look at it again. Yet there are important reasons why a fracture should be looked at even frequently. In the first place, the swelling that is present more or less when the splints are first put on goes down, and as a result the splints and bandages become loose. The result of this, of course, may be re-displacement of the fracture. This occurs, not infrequently, in fractures of the lower end of the radius. In consequence of the above-mentioned fact, I am in the habit of taking down all fractures on the second or third day after the injury, and readjusting the apparatus. After a while, if the apparatus be kept on long, wasting of the muscles of the limb occurs, and again the apparatus becomes loose and useless, and requires readjustment. It is evident, therefore, that to treat such cases properly, any apparatus that is applied may have to be taken off and readjusted two or three times during the course of treatment. If the circumstances of the fracture require this, the necessities of the injury to the joint demand more, and if the apparatus, whatever it be, can be taken off two or three times, with benefit to the fracture, why not more frequently for the sake of the joint? As has been already said, our great object should be to provide our patients with sound and useful limbs as soon as possible—in the shortest possible time. Now we know that if a bone be broken a certain number of weeks must elapse before that bone will be firmly enough united to allow of the limb being used (varying according to the size of the bone and the age of the patient). At the end of the necessary period, let us suppose, the bone is united but the limb is not fit for use. Why? Because it is stiff. And why is it stiff? Because tendons have become adherent to their sheaths, ligaments have become thickened and contracted, and adhesions have formed. These adhesions have to be broken down, ligaments stretched, and stiffness generally removed by a course of massage and passive movement which occupies another week or two. And not unfrequently even then the joint remains stiff and comparatively useless for months, or even permanently.

I am sure it is quite within the experience of most surgeons that many persons never recover the use of their limbs perfectly after fracture, sometimes from persistent deformity, but more frequently from stiffness of the neighbouring joint. This should be prevented, and in the majority of cases I am sure it can be prevented, by the judicious employment of massage and passive movement applied, not at the end of the treatment of the case, but from the beginning. In short, as I have tried to show, instead of treating the fracture first, and the damaged and stiffened joint afterwards, we should treat both at the same time, and from the beginning, and so save time for our patients. Why should a case of fractured radius have to endure splints for three weeks, and then have to go through a course of massage for perhaps six weeks afterwards, when three weeks of treatment might be sufficient? Why should a case of Pott's fracture remain in bed for a month or six weeks, and then have to hobble about for another month or six weeks, when he might be made able to walk with freedom and comfort in about half that time? Am I exaggerating, or stating the case in regard to our former treatment of these cases too darkly? I am sure I am not. I know that many cases of Colles' fracture have stiff wrists and useless fingers. Many cases of fractures at the elbow result in anchylosis (more or less) of that joint, and in some resection is necessary. Many cases of fracture near the ankle result in lameness which may persist for a long time, but fortunately, ultimately disappears or is reduced to a tolerable minimum, in a considerable number of instances. Why does an ankle so often recover function while an elbow more frequently becomes stiff? May it not be, as my friend Dr. Laing holds, because the one must be used, while the other may be kept rigid for a long time without very great inconvenience?

Now, the method of treatment which I advocate here as the most rational and the best fitted to give the patient a useful limb in the shortest possible time is what I have indicated, viz., the employment of massage and passive movement from the very beginning, along with rest and retentive apparatus.

I would describe a typical case thus. Let the fracture be carefully placed in proper position at once, and retained in position by well-padded splints or some other suitable apparatus, the patient, of course, being placed in bed if the lower limb be the one injured. Then after one, or at most two days, let the apparatus be removed, the limb above the injury and the joint be gently stroked upwards and very softly massaged, and the joint very slightly and gently moved, and the apparatus carefully readjusted, special care being taken to see that the parts are in proper position This procedure should be repeated daily for a few days (or every second day, if the patient complains of the manipulations), beginning with a few minutes of massage, and gradually extending the performance to ten minutes or a quarter of an hour. Then the massage may be made gradually more prolonged, and the passive movements more extensive, and consequently the operation need not be performed daily, but every second or third day. Of course, I need hardly say that care—great care—must be taken not to move the fractured surfaces of bone when this is being done, and also that whatever apparatus is employed in the treatment of the fracture must be one that can be easily taken off and on, and that without displacing the fractured ends of bone. For example, the figure-of-eight bandage for the elbow and a box or Clines' splints for the leg.

It will be evident that what I recommend is, that the treatment of a fracture and of a sprain should be combined in these cases. Keeping this in mind, viz., that there is a double injury in these cases, the question comes to be, which is the more important? Formerly we considered the fracture the more important; surgeons were afraid of non-union. But really, if we consult our experience, we find that stiff joints are far more common than ununited fractures. Is the injured joint, then, the more important injury? I would not say so. It seems to me that the safest course is to consider, as I have said, that we have a double injury to deal with, and that the treatment of the two has to be carried on at the same time. Both are to be considered and treated together. We cannot neglect either without loss and damage to the patient. Which of the injuries, if any, is to be considered the more important must be settled in each case according to circumstances, regard being always paid to the object of our treatment, viz., to produce a useful limb in the shortest possible time.

